# ITPKA induces cell senescence, inhibits ovarian cancer tumorigenesis and can be downregulated by miR-203

**DOI:** 10.18632/aging.202880

**Published:** 2021-04-20

**Authors:** Wang Shaosheng, Wang Shaochuang, Fan Lichun, Xie Na, Zhao Xiaohong

**Affiliations:** 1 Maternity Service Center of Pengzhou Maternal & Children Health Care Hospital, Chengdu, Sichuan Province 611930, People’s Republic of China; 2 Department of Hepatobiliary and Pancreatic Surgery, Huai’an First People’s Hospital, Nanjing Medical University, Huai'an 223300, Jiangsu Province, People’s Republic of China; 3 Hainan Maternal and Children’s Medical Center, Haikou 570206, Hainan Province, People’s Republic of China; 4 Department of Pathology, The Affiliated Hospital of Hainan Medical University, Haikou 571101, Hainan Province, People’s Republic of China

**Keywords:** ovarian cancer, ITPKA, cell senescence, MDM2

## Abstract

Overcoming senescence is a feature of ovarian cancer cells; however, the mechanisms underlying senescence regulation in ovarian cancer cells remain largely unknown. In this study, we found that ITPKA was downregulated in ovarian cancer samples, and the lower expression correlated with poor survival. Overexpression of ITPKA inhibited the anchorage-independent growth of ovarian cancer cells and induced senescence. However, knockdown of ITPKA promoted the anchorage-independent growth of ovarian cancer cells and inhibited senescence. Mechanistically, ITPKA was found to interact with MDM2, which stabilized P53, an essential regulator of senescence. Moreover, ITPKA was negatively regulated by miR-203, a microRNA that has been previously reported to be upregulated in ovarian cancer. Taken together, the results of this study demonstrated the tumor suppressive roles of ITPKA in ovarian cancer and provided a good explanation for the oncogenic roles of miR-203.

## INTRODUCTION

Ovarian cancer is a common malignancy for women worldwide [1, 2], and although surgical resection, chemotherapy, radiotherapy and other therapeutic strategies have been used to treat this malignancy, the outlook for this disease is still not optimistic [3, 4]. A better understanding of the molecular mechanism guiding this disease would definitely benefit therapies.

Senescence is considered a state that inhibits tumor growth [5, 6]. P53, P21, P27 and P16 are the key regulators of senescence [7–9]. MDM2 is an E3 ubiquitin ligase that promotes the degradation of P53 to tightly control P53 protein levels [10, 11]. Numerous studies have shown that the induction of senescence inhibits the growth, colony formation and tumorigenesis of ovarian cancer [12, 13]. Therefore, the identification of novel regulators of senescence might provide therapeutic targets for ovarian cancer.

Inositol-trisphosphate 3-kinase A (ITPKA) promotes the motility of cancer cells by controlling the dynamics of the cytoskeleton [14–17]. Although previous studies have shown that ITPKA is expressed in a broad range of tumor types and that ITPKA gene body methylation inhibits its expression, thus serving as a novel and potential biomarker for early cancer detection, the expression pattern of ITPKA in ovarian cancer remains unknown [14–17]. The biological functions of ITPKA in lung cancer and breast cancer have attracted considerable attention. In lung cancer, ITPKA has been reported as a marker of growth pattern-specific gene signatures in pulmonary adenocarcinoma [14, 18, 19], and ITPKA exhibits oncogenic activity in lung cancer cells by regulating Ins(1,4,5)P_3_-mediated calcium release and cytoskeletal dynamics [20]. In addition, inositol-trisphosphate 3-kinase A (ITPKA) was a significantly enriched differentially expressed gene associated with the inositol phosphate metabolism pathway in glioma cells [21, 22]. However, whether the expression and function of ITPKA are context-dependent remains unknown.

Our previous study demonstrated the upregulation of miR-203 in ovarian cancer [23]. However, the target genes of miR-203 in ovarian cancer remain largely unknown. In this study, we examined the expression pattern and functions of ITPKA in ovarian cancer and investigated the underlying molecular mechanisms. Moreover, we explored the regulation of ITPKA by miR-203 in ovarian cancer.

## RESULTS

### ITPKA inhibited the colony formation of cancer cells and induced cell senescence

**Figure 1:**
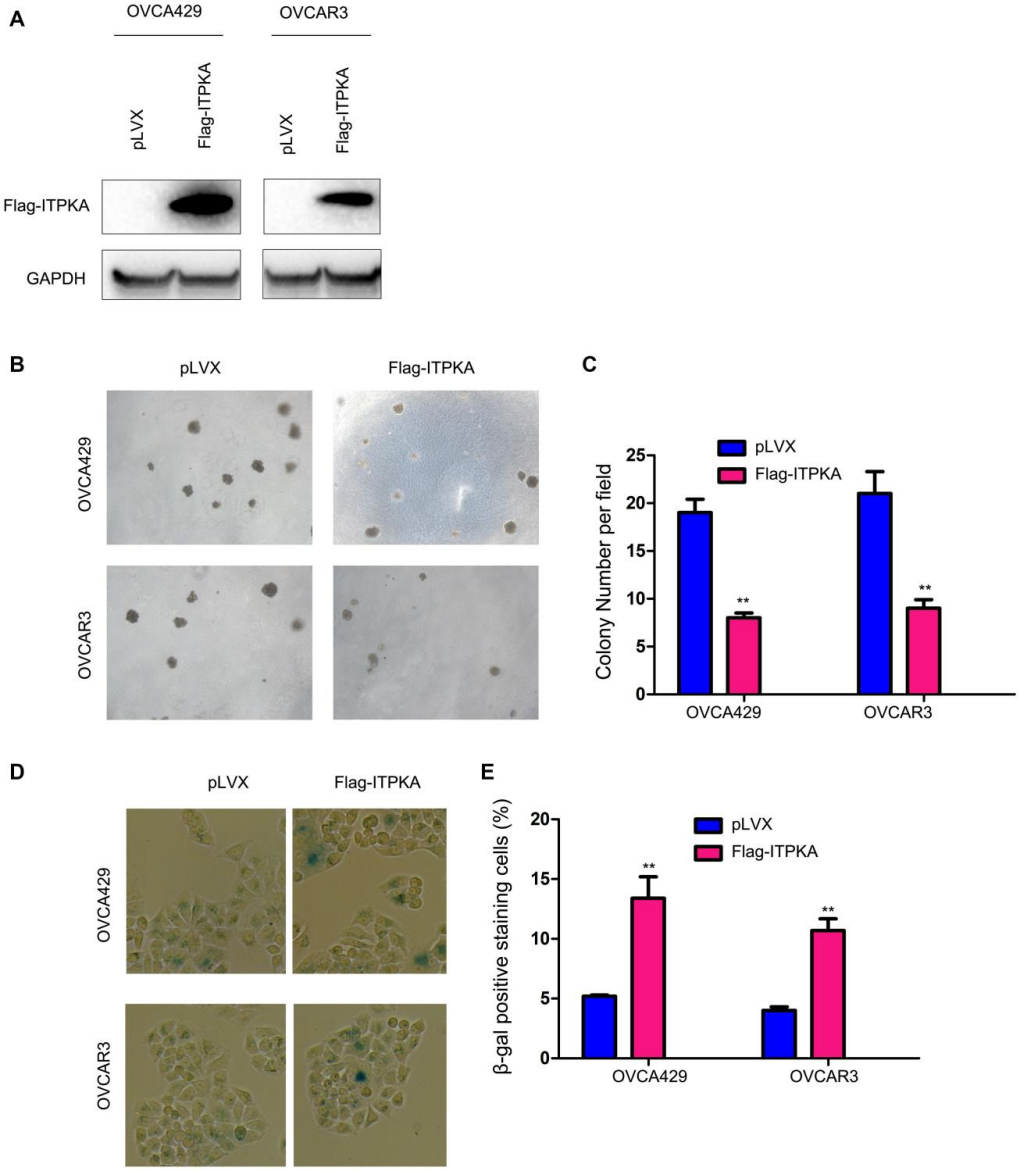
**ITPKA inhibited colony formation and induced cell senescence in ovarian cancer.** (**A**) Overexpression of ITPKA in OVCAR3 and OVCA429 cells. Cells were transfected with pLVX plasmids or pLVX-Flag-ITPKA plasmids. After selection, the resistant cells were examined for the expression of Flag-tagged ITPKA. (**B**) Effects of ITPKA on the colony formation of OVCAR3 and OVCA429 cells were examined using a soft agar assay. (**C**) Statistical analysis of (**B**). (**D**) Effects of ITPKA on the senescence of OVCAR3 and OVCA429 cells were examined using β-gal staining. (**E**) Statistical analysis of (**D**). ^**^P < 0.01.

To investigate the functions of ITPKA in ovarian cancer, we transfected the ovarian cancer cell lines OVCA429 and OVCAR3 with a vector (Flag-ITPKA) to overexpress ITPKA (Figure 1A). The effects of ITPKA expression on colony formation were examined using a soft agar assay. As shown in Figure 1B–1C, forced expression of ITPKA inhibited the anchorage-independent growth of OVCA429 and OVCAR3 cells on soft agar. Moreover, β-gal staining revealed that ITPKA induced cell senescence (Figure 1D–1E).

**Figure 2:**
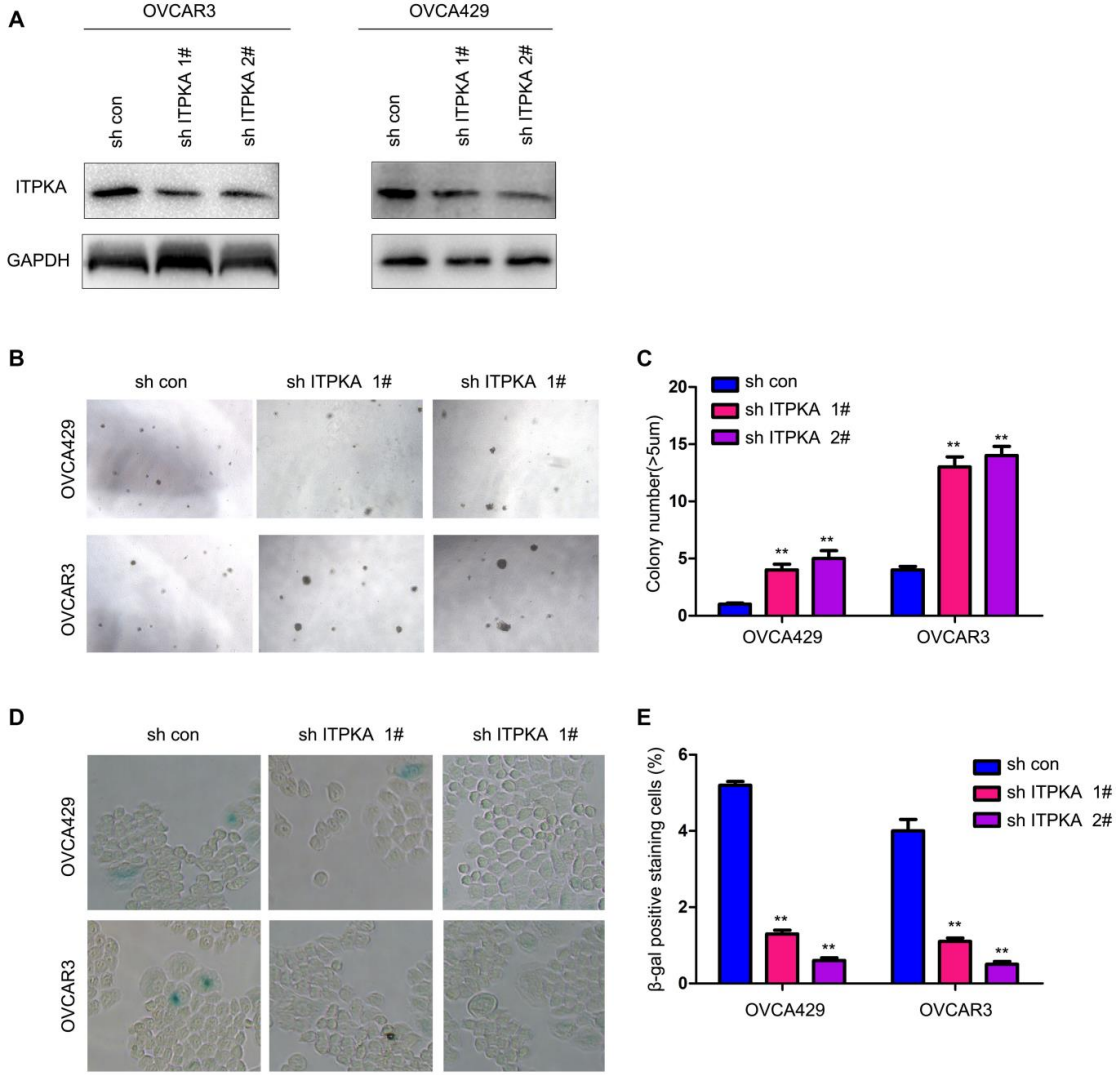
**Knocking down ITPKA promoted colony formation and inhibited cell senescence in ovarian cancer.** (**A**) Knockdown of ITPKA in OVCAR3 and OVCA429 cells. Cells were infected with a lentivirus for 8 hours. After selection, the resistant cells were examined for ITPKA expression. (**B**) Effects of ITPKA knockdown on the colony formation of OVCAR3 and OVCA429 cells were examined using a soft agar assay. (**C**) Statistical analysis of (**B**). (**D**) Effects of ITPKA knockdown on the senescence of OVCAR3 and OVCA429 cells were examined using β-gal staining. (**E**) Statistical analysis of (**D**). ^**^P < 0.01.

To further understand the functions of ITPKA in the progression of ovarian cancer, the expression of ITPKA was knocked down in OVCA429 and OVCAR3 cells (Figure 2A). Knockdown of ITPKA expression enhanced the anchorage-independent growth of ovarian cancer cells and inhibited cell senescence (Figure 2B–2E). Taken together, these results demonstrated that ITPKA inhibited the colony formation of cancer cells and induced cell senescence.

### ITPKA inhibited the ovarian cancer cell tumorigenesis *in vivo*

**Figure 3:**
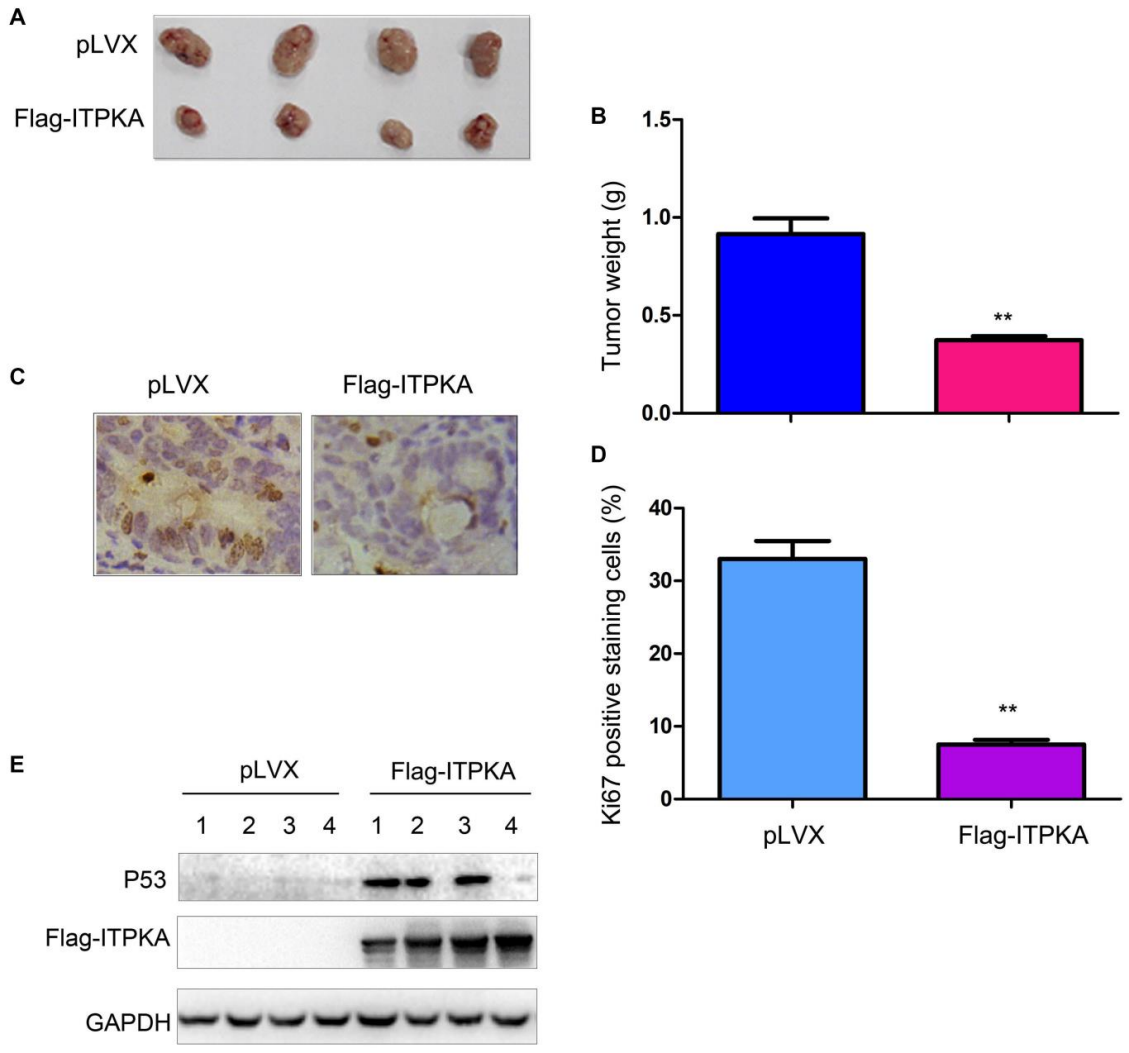
**ITPKA inhibited the tumorigenicity of ovarian cancer.** (**A**) Gross images of the tumors formed by OVCAR3 control cells and OVCAR3/Flag-ITPKA cells in nude mice. (**B**) Tumor weight as shown in (**A**). (**C**) Immunostaining was performed to examine Ki67 expression. (**D**) Statistical analysis of (**C**). (**E**) Expression of P53 and Flag-ITPKA in the tumors was examined via western blot. ^**^P < 0.01.

To extend our study to an *in vivo* system, we injected OVCAR3 control cells (OVCAR3/pLVX) and ITPKA-overexpressing cells (OVCAR3/Flag-ITPKA) into nude mice. Consistent with the *in vitro* study, overexpression of ITPKA impaired the tumorigenicity of OVCAR3 cells, as demonstrated by the tumor weight values (Figure 3A–3B). Examination of the tumors using immunohistochemistry (IHC) for Ki67 revealed less Ki67 staining in the tumors formed by the OVCAR3/Flag-ITPKA cells (Figure 3C–3D) compared to that in the control cells, suggesting that ITPKA inhibited cell proliferation. Moreover, P53 protein levels were increased in the tumors formed by OVCAR3/Flag-ITPKA cells (Figure 3E).

### ITPKA interacted with MDM2 and stabilized P53

**Figure 4:**
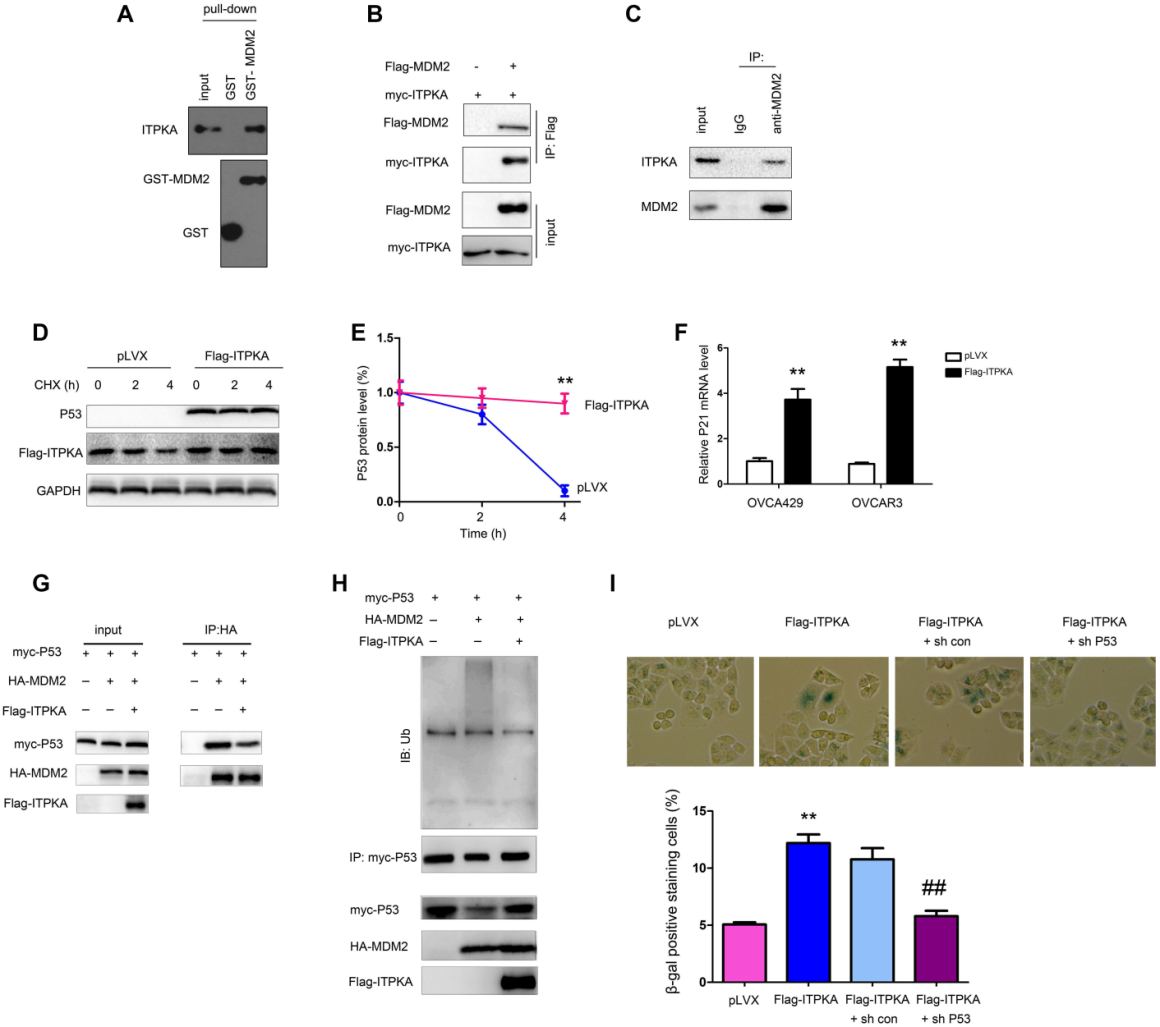
**ITPKA interacted with MDM2.** (**A**) GST pull-down assays were performed to examine the interaction between the fusion proteins GST-MDM2 and ITPKA. OVCAR3 cell lysates were used. (**B**) Immunoprecipitation assay was performed to examine the interaction between flag-tagged MDM2 and myc-tagged ITPKA. Flag-MDM2 and myc-ITPKA were transfected into OVCAR3 cells. Forty-eight hours later, the cells were harvested, and immunoprecipitation assays were performed using an anti-Flag antibody. (**C**) Immunoprecipitation assay was performed to examine the interaction between endogenous MDM2 and ITPKA. Protein from OVCAR3 cells was harvested, and immunoprecipitation assays were performed using an anti-MDM2 antibody. (**D**–**E**) Stability of P53 was examined after the cells were treated with CHX at the indicated time points. (**F**) mRNA level of P21 was examined using q-PCR. (**G**) Immunoprecipitation was performed to examine the interaction between P53 and MDM2 in OVCAR3 cells. (**H**) Ubiquitination of P53 was examined in OVCAR3 cells. (**I**) P53 knockdown abolished the function of ITPKA in cell senescence. ^**^P < 0.01; ^##^*P* < 0.01.

We next explored the molecular mechanisms through which ITPKA inhibited the tumorigenicity of ovarian cancer cells. Considering the induction of cell senescence by ITPKA, a GST pull-down assay was performed, and then the interactions between ITPKA and a major component of senescence-related pathways were examined. Interestingly, in the GST pull-down assay, the interaction between the fusion protein GST-MDM2 and ITPKA was detected (Figure 4A), which was further demonstrated by an immunoprecipitation assay (Figure 4B–4C). Next, the effects of ITPKA on the stability of P53 were assessed. ITPKA induced the accumulation of P53 upon CHX treatment as well as the expression of P21, the downstream target of P53 (Figure 4D–4F). Therefore, ITPKA interacted with MDM2 and stabilized P53 to inhibit cell growth and induce senescence.

To further investigate the mechanism in detail, we examined the ubiquitination of P53. Overexpression of ITPKA inhibited the interaction of MDM2 and P53 and the ubiquitination of P53 (Figure 4G–4H). In the functional study, knockdown of P53 blocked cell senescence induced by ITPKA (Figure 4I), indicating that the functions of ITPKA were dependent on P53.

### ITPKA was downregulated in ovarian cancer and negatively regulated by miR-203

**Figure 5:**
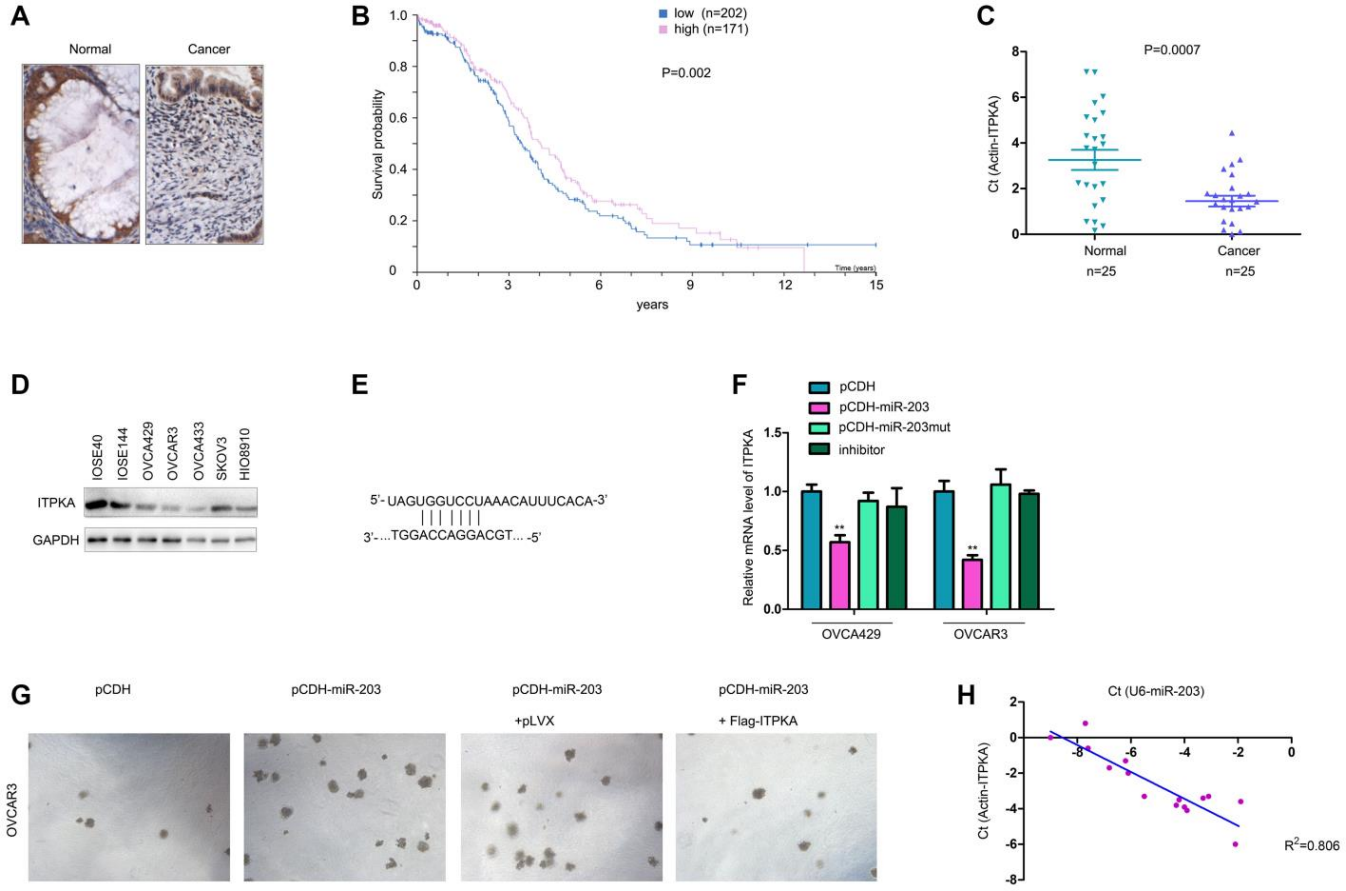
**ITPKA was downregulated in ovarian cancer and negatively regulated by miR-203.** (**A**) IHC was performed to examine the protein levels in ovarian cancer and normal tissues. (**B**) GEPIA database mining was performed to determine the correlation between ITPKA expression and survival. (**C**) q-PCR was performed to examine the mRNA levels of ITPKA in ovarian cancer samples and normal tissues. (**D**) Western blotting was performed to examine the ITPKA protein level in ovarian cancer cell lines and normal ovarian epithelial cell lines (IOSE80 and IOSE144). (**E**) Illustration of miR-203 and ITPKA. (**F**) Effects of miR-203, mutant miR-203 and inhibitor on the expression of ITPKA were examined using q-PCR. (**G**) Soft agar assays were performed to examine the effects of ITPKA on the tumorigenicity of OVCAR3 cells. (**H**) Expression of miR-203 and ITPKA in ovarian cancer samples. ^**^P < 0.01.

The expression of ITPKA was evaluated in ovarian cancer samples. IHC staining clearly showed the downregulation of ITPKA in ovarian cancer tissues (Figure 5A). Mining the GEPIA database showed that lower ITPKA expression was associated with poor survival (Figure 5B). Consistent with this observation, downregulation of ITPKA mRNA was observed in ovarian cancer tissues (Figure 5C). Moreover, the protein level of ITPKA was higher in the normal ovarian epithelial cell lines (IOSE80 and IOSE144) (Figure 5D). These results confirmed the downregulation of ITPKA in ovarian cancer.

In a previous study, we demonstrated that miR-203 is upregulated in ovarian cancer. Therefore, we tested whether miR-203 inhibited the expression of ITPKA. As shown in Figure 5E–5F, miR-203 negatively regulated the mRNA level of ITPKA in OVCA429 and OVCAR3 cells (Figure 5E–5F). Moreover, the ability of miR-203 to promote colony formation of OVCAR3 cells was abolished by ITPKA (Figure 5G). In addition, we found a negative association between the expression of miR-203 and the mRNA levels of ITPKA (Figure 5H). In summary, ITPKA was downregulated in ovarian cancer and negatively regulated by miR-203.

## DISCUSSION

Although several studies have shown that ITPKA is upregulated in some cancer types [14], our study clearly demonstrated that ITPKA is downregulated in ovarian cancer, suggesting that the expression of ITPKA in cancer is dependent on the context of the tumor. Moreover, we have provided evidence that ITPKA possesses tumor suppressor activity in ovarian cancer cells. ITPKA inhibited ovarian cancer cell colony formation and tumorigenesis and induced cell senescence. Moreover, we have provided evidence that ITPKA stabilizes P53. These results confirmed the tumor suppressive roles of ITPKA in ovarian cancer.

One interesting finding of this study is the induction of cell senescence by ITPKA. To our knowledge, this is the first report of the role of ITPKA in cell senescence. Numerous studies have demonstrated that cancer cells can overcome senescence and that senescence induction might be a therapeutic strategy for treating ovarian cancer [24, 25]. In this study, ITPKA was shown to induce senescence possibly by antagonizing the functions of MDM2.

Another interesting aspect of this study is the negative regulation of ITPKA by miR-203. In our previous work, we showed that miR-203 was upregulated in ovarian cancer and associated with poor survival [23]. However, the downstream targets of miR-203 have not been fully elucidated. In this study, we showed that ITPKA expression was inhibited by miR-203 and that the functions of miR-203 were abolished by overexpressing ITPKA. These results indicate that downregulation of ITPKA mediates the biological functions of miR-203 in ovarian cancer.

In fact, the roles of miR-203 in ovarian cancer are controversial. Although two studies have reported that miR-203 inhibits ovarian tumor metastasis by targeting BIRC5, attenuating the TGFβ pathway, and inhibiting epithelial to mesenchymal transition [26, 27], one of these studies did not examine the expression of miR-203 in clinical ovarian cancer samples, and the other study was performed using human ovarian serous carcinoma tissues. Moreover, both studies focused on the metastasis of ovarian cancer. These observations only supported the notion that miR-203 promoted metastasis and did not indicate that miR-203 was a tumor suppressor in primary tumor formation.

The oncogenic roles of miR-203 in ovarian cancer are supported by several critical studies. A multi-institutional study demonstrated that miR-203 was an independent molecular predictor of poor prognosis and outcome in ovarian cancer [28], which was consistent with our study showing that upregulation of microRNA-203 is associated with advanced tumor progression and poor prognosis in epithelial ovarian cancer [23]. Moreover, we have shown the molecular mechanism by which miR-203 enhances the glycolytic pathway [29]. Taken together, we think that miR-203 (oncogene or tumor suppressor) might play different roles at different stages (primary and metastasis) of cancer.

The induction of senescence by IPTKA suggested that restoring the expression of ITPKA (using an inhibitor of miR-203) and treatment with an agonist of ITPKA would be promising strategies for ovarian cancer therapy. Additionally, in future studies, it is necessary to verify the effects of ITPKA on senescence using an ovarian cancer mouse model.

In summary, this study has demonstrated the tumor suppressive roles of ITPKA in ovarian cancer, which suggests that activation of ITPKA might be beneficial for the treatment of ovarian cancer.

## MATERIALS AND METHODS

### Cell culture

The ovarian cancer cell lines OVCAR3, OVCA429 and HEK293T were purchased from the cell bank of the Chinese Academy of Science (Shanghai, China). Cells were placed in an incubator at 37°C with 5% CO_2_ and maintained in DMEM containing 10% FBS and antibiotics.

### Clinical samples

Clinical samples were collected from the Hainan Maternal and Children’s Medical Center after obtaining patients’ written informed consent. The pathology of each sample was confirmed by two pathologists.

### Plasmids

The coding sequence of ITPKA (NM002220.3) was cloned into the pLVX (containing the Flag tag) and pcDNA3.1 (containing the myc tag) vectors. The coding sequence of MDM2 (NM002392.6) was cloned into pGEX-4T-1 (containing the GST tag) to produce the fusion protein GST-MDM2. The sh RNA sequences were inserted into the cassette of the pLKO.1 vector. The sequences for sh ITPKA were as follows: sh ITPKA #1, 3′-aacgtgcagctggaagcgggc-5′; and sh ITPKA #2, 3′- aagctacctgcagctgcagga-5′.

### qPCR

Twenty microliters of Hieff qPCR SYBR^®^ Green Master Mix (No Rox Plus) was used for the amplification reaction. The system included 10 μL of PCR MIX, 0.4 μL of 10 μm forward primer, 0.4 μL of 10 μm reverse primer, and 3 μL of cDNA template, and it was supplemented with ddH_2_O to a volume of 20 μL. All reactions were performed in duplicate and detected on a Thermo Scientific™ PikoReal™ Real-Time PCR Detection System. The reaction conditions were as follows: 95°C for 3 minutes; 40 cycles of 94°C for 30 seconds and 60°C for 30 seconds; 95°C for 15 s; 60°C for 60 s; and 95°C for 15 s. The melt curve was plotted to determine the specificity of amplification. The forward primer of ITPKA was 5′-CTTCGACGGACCTTGTGTG-3′, and the reverse primer was 5′-CACCGCCAGCATTTTCTTGT-3′. The forward primer of P21 was 5′- TGTCCGTCAGAACCCATGC-3′, and the reverse primer was 5′- AAAGTCGAAGTTCCATCGCTC-3′.

### Cell transfection

For virus packaging, HEK293T cells were transfected using Lipofectamine 2000 according to the manufacturer’s instructions. The virus was harvested 24 and 48 hours after transfection. After filtration, the virus was used to infect OVCAR3 and OVCA429 cells. Forty-eight hours later, the infected OVCAR3 and OVCA429 cells were selected by treatment with puromycin for 1 week. Then, the resistant cells were pooled and the expression of ITPKA was examined.

### Soft agar assay

A bottom layer was generated that contained 0.5% agarose and 10% FBS in DMEM, and it was used to coat a 12-well plate. The upper layer in the 12-well plates contained 0.35% agarose and 10% FBS in DMEM. Then, 2 × 10^3^ cells were suspended in the upper layer. Colonies that formed were photographed and counted after 14 days of incubation. All experiments were performed at least three times.

### β-Gal staining

Cells were plated in 24-well plates at a density of 2 × 10^5^/well. Twenty-four hours later, the senescence of the cells was examined using a kit (Beyond, Shanghai) according to the instructions.

### Immunohistochemistry

IHC was performed as previously described [30]. Briefly, the tissues were treated with xylol to remove paraffin. Then, the sections were treated with ethanol (from a 100% to 75% gradient). After extensive washing with 0.1 M PBS, antigen recovery was performed by incubating tissues in 1 M citrate sodium solution (pH 6.0) at 100°C for 20 minutes. Then, the tissues were blocked with 5% BSA at room temperature for 1 hour. Each primary antibody (1:200) was incubated with tissues overnight at 4°C. The next day, the tissues were washed with PBS before incubating again with the secondary antibody at room temperature for 1 hour. The tissues were then developed using DAB and counterstained with hematoxylin.

### GST-MDM2 fusion protein

The coding sequence of MDM2 (NM002392.6) was cloned into pGEX-4T-1 (containing the GST tag). BL21 competent cells were transformed with pGEX-4T-1 empty vectors or pGEX-4T-1 vectors containing the MDM2 coding sequence. An overnight culture was set up in 50 ml 2*TY with 150 mg/ml ampicillin. The next day, 5 ml of the overnight culture was seeded in 500 ml 2*TY with 150 mg/ml ampicillin and grown at 37°C to an A600 of 0.6–0.8, and then the culture was induced with 0.1 mM to 2 mM IPTG and grown for another 3 hours at 37°C. The cells were pelleted by centrifugation at 3000 g and 4°C for 10 min. The medium was decanted, and then the cells were resuspended and washed in 30 ml ice-cold PBS, transferred to a 40-ml Oak Ridge tube and centrifuged at 3000 g and 4°C for 10 min. The supernatant was discarded and the pellet was resuspended in 10 ml of ice-cold STE buffer. Then, 100 ml of freshly prepared lysozyme solution was added to the suspension and incubated on ice for 15 min. Immediately before sonication, 100 ml of 1 M DTT and 1.4 ml of 10% sarkosyl were added and mixed thoroughly by inversion and sonicated for a total of 1 min. Debris was pelleted via centrifugation at 16000 rpm for 20 min on an SS34 rotor. The supernatant was transferred to a 50-ml conical tube, and the pellet was discarded. Then, 4 ml of 10% Triton X-100 and STE buffer to a volume of 20 ml were added. The effective concentrations of sarkosyl and Triton X-100 were 0.7% and 2%, respectively. The suspension was incubated at room temperature for 30 min and poured into a 1 ml bed of prepared glutathione Sepharose in PBS. Incubation was performed at room temperature for 30 min to 1 hour with agitation. The beads were washed with 150 ml of PBS, resuspended in 5 ml of PBS and poured into a dispo-column. The beads were washed in a 50-ml conical tube with an additional 5 ml of PBS and pooled with the first 5 ml in the dispo-column. The fusion protein was eluted with 10* 1 ml fractions of elution buffer. The desired fractions were determined via SDS-PAGE.

### Western blot

Cells were harvested with RIPA buffer. The protein concentration was determined using BCA. Then, SDS-PAGE was performed, and the proteins were transferred to a PVDF membrane. After blocking with 5% BSA at room temperature for 1 hour, the membrane was incubated with the primary antibody for 4 hours and sequentially with the secondary antibody for 1 hour. Then, the signals were examined using an ECL kit. The following antibodies were used: anti-ITPKA (Sigma, HPA040454), anti-GAPDH (Santa Cruz, sc-47724), anti-GST (Santa Cruz, sc-138), anti-Flag (Proteintech, 80010-1-RR), anti-Myc (Santa Cruz, sc-40), anti-tubulin (Santa Cruz, sc-166729), anti-HA (Proteintech, 51064-2-AP), anti-P53 (CST, 48818), anti-MDM2 (CST, 86934), and anti-ubiquitin (CST, 3936).

### GST pull-down

Fusion proteins were purified using Sepharose 4B beads, which were incubated with lysates from OVCAR3 cells for 4 hours. The proteins that were pulled down were separated by SDS-PAGE.

### Immunoprecipitation

Cells were transfected with the indicated vectors with Lipofectamine 2000. Forty-eight hours later, the protein was harvested from the cells using RIPA buffer. After centrifugation, the supernatant was incubated with the indicated antibody for 4 hours. Then, protein A beads were added and incubated for another 4 hours. After extensive washing, the immunoprecipitated proteins were examined using western blots.

### Subcutaneous tumor formation in nude mice

Eight-week-old male nude mice (SLAC, Shanghai) were randomly divided into two groups, with 4 mice each. The mice in one group were injected with OVCAR3 cells (1 × 10^6^ cells/site) containing pLVX, and the mice in the other group were injected with OVCAR3 cells (1 × 10^6^ cells/site) containing Flag-ITPKA. The mice were sacrificed after 5 weeks, the tumor tissues were excised and weighed, and ITPKA expression was evaluated via western blot. This study was approved by the ethical committee of the Hainan Maternal and Children’s Medical Center and complied with the ethical regulations of the ethical committee of the Hainan Maternal and Children’s Medical Center.

### Statistical analysis

All sample sizes were sufficient to ensure proper statistical analysis. Data are represented as the means ± SEM of at least three experiments. Statistical analyses were performed using GraphPad Prism 6 software, version 6 (GraphPad Software, Inc.). Statistical significance was calculated using Student’s two-tailed unpaired *t*-tests. The log-rank (Mantel-Cox) test was used for survival comparisons; ns, not significant (*P* > 0.05); ^*^P < 0.05; ^**^P < 0.01; ^***^P < 0.001; ^****^P < 0.0001.

### Ethics approval and consent to participate

All experimental protocols were approved by the Hainan Maternal and Children's Medical Center Institutional Committee. Informed consent was obtained from all subjects. The study was reviewed and approved by the China National Institutional Animal Care and Use Committee.
